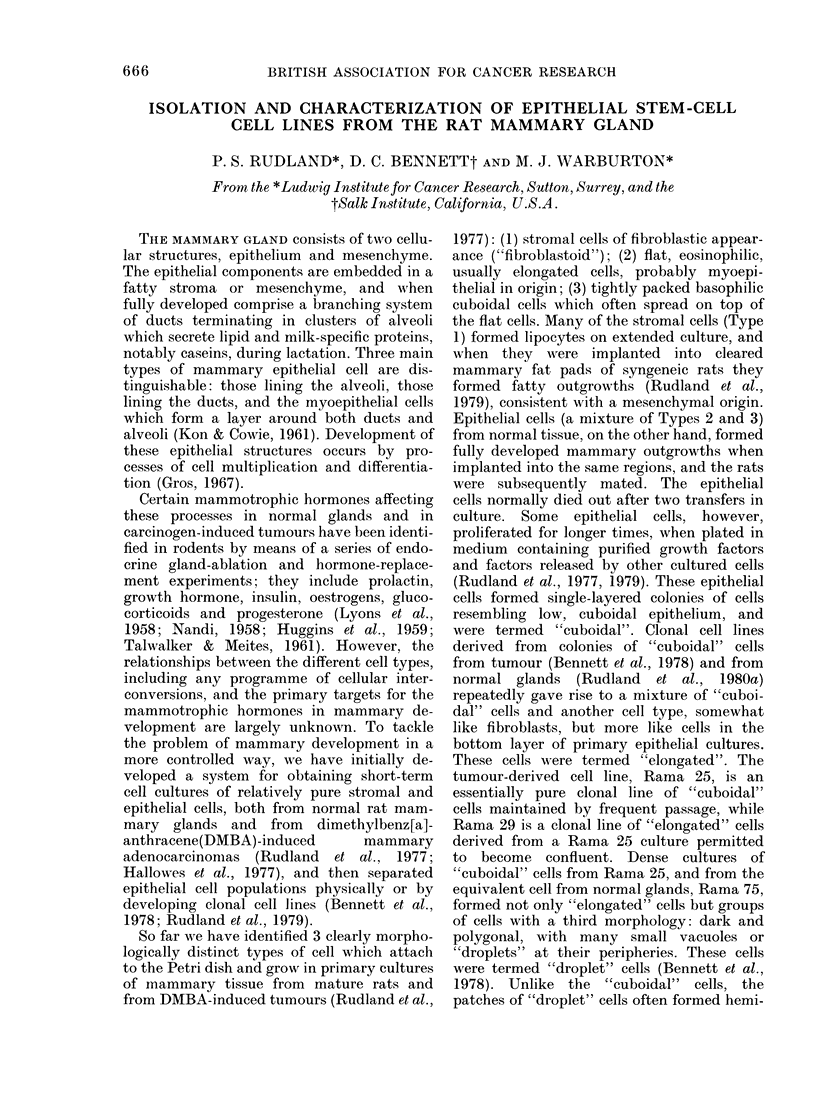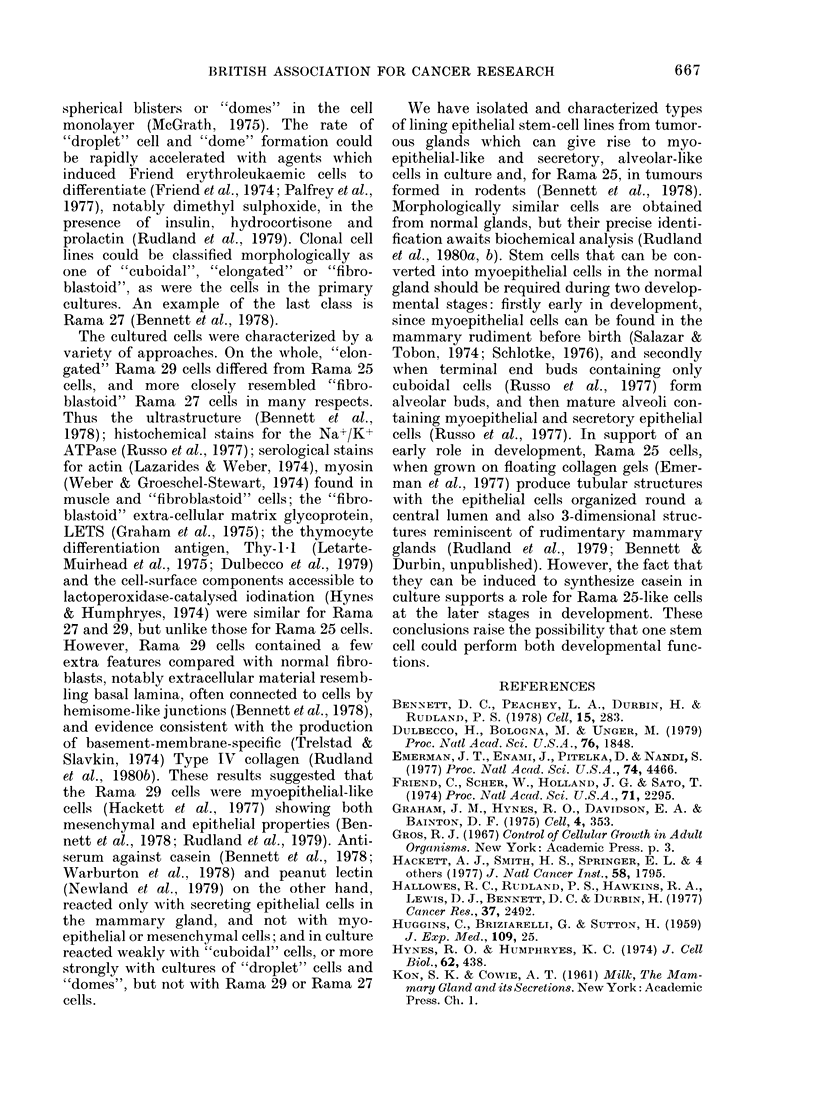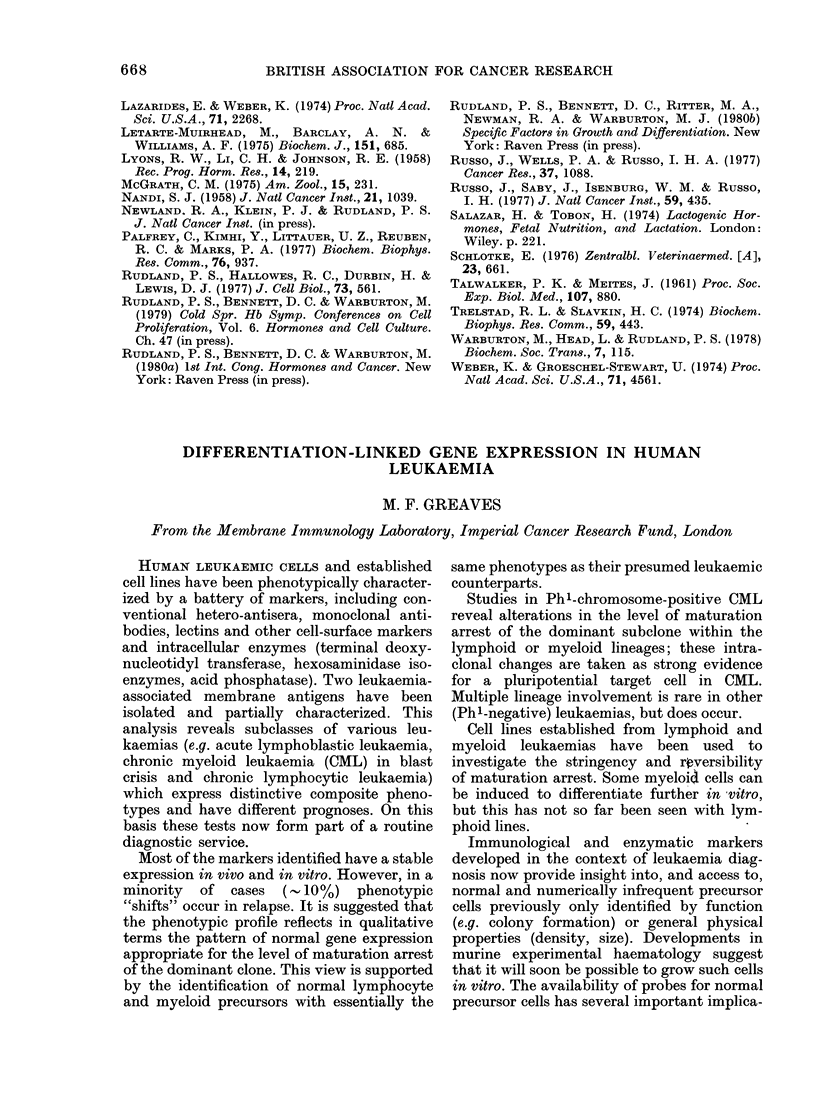# Isolation and characterization of epithelial stem-cell cell lines from the rat mammary gland.

**DOI:** 10.1038/bjc.1980.119

**Published:** 1980-04

**Authors:** P. S. Rudland, D. C. Bennett, M. J. Warburton


### ISOLATION AND CHARACTERIZATION OF EPITHELIAL STEM-CELL CELL LINES FROM THE RAT MAMMARY GLAND

P. S. RUDLAND*, D. C. BENNETT† AND M. J. WARBURTON*

*From the *Ludwig Institute for Cancer Research, Sutton, Surrey, and the †Salk Institute, California, U.S.A.*

THE MAMMARY GLAND consists of two cellular structures, epithelium and mesenchyme. The epithelial components are embedded in a fatty stroma or mesenchyme, and when fully developed comprise a branching system of ducts terminating in clusters of alveoli which secrete lipid and milk-specific proteins, notably caseins, during lactation. Three main types of mammary epithelial cell are distinguishable: those lining the alveoli, those lining the ducts, and the myoepithelial cells which form a layer around both ducts and alveoli (Kon & Cowie, 1961). Development of these epithelial structures occurs by processes of cell multiplication and differentiation (Gros, 1967).

Certain mammotrophic hormones affecting these processes in normal glands and in carcinogen-induced tumours have been identified in rodents by means of a series of endocrine gland-ablation and hormone-replacement experiments; they include prolactin, growth hormone, insulin, oestrogens, glucocorticoids and progesterone (Lyons *et al.*, 1958; Nandi, 1958; Huggins *et al.*, 1959; Talwalker & Meites, 1961). However, the relationships between the different cell types, including any programme of cellular interconversions, and the primary targets for the mammotrophic hormones in mammary development are largely unknown. To tackle the problem of mammary development in a more controlled way, we have initially developed a system for obtaining short-term cell cultures of relatively pure stromal and epithelial cells, both from normal rat mammary glands and from dimethylbenz[a]-anthracene(DMBA)-induced mammary adenocarcinomas (Rudland *et al.*, 1977; Hallowes *et al.*, 1977), and then separated epithelial cell populations physically or by developing clonal cell lines (Bennett *et al.*, 1978; Rudland *et al.*, 1979).

So far we have identified 3 clearly morphologically distinct types of cell which attach to the Petri dish and grow in primary cultures of mammary tissue from mature rats and from DMBA-induced tumours (Rudland *et al.*, 1977): (1) stromal cells of fibroblastic appearance ("fibroblastoid"); (2) flat, eosinophilic, usually elongated cells, probably myoepithelial in origin; (3) tightly packed basophilic cuboidal cells which often spread on top of the flat cells. Many of the stromal cells (Type 1) formed lipocytes on extended culture, and when they were implanted into cleared mammary fat pads of syngeneic rats they formed fatty outgrowths (Rudland *et al.*, 1979), consistent with a mesenchymal origin. Epithelial cells (a mixture of Types 2 and 3) from normal tissue, on the other hand, formed fully developed mammary outgrowths when implanted into the same regions, and the rats were subsequently mated. The epithelial cells normally died out after two transfers in culture. Some epithelial cells, however, proliferated for longer times, when plated in medium containing purified growth factors and factors released by other cultured cells (Rudland *et al.*, 1977, 1979). These epithelial cells formed single-layered colonies of cells resembling low, cuboidal epithelium, and were termed "cuboidal". Clonal cell lines derived from colonies of "cuboidal" cells from tumour (Bennett *et al.*, 1978) and from normal glands (Rudland *et al.*, 1980*a*) repeatedly gave rise to a mixture of "cuboidal" cells and another cell type, somewhat like fibroblasts, but more like cells in the bottom layer of primary epithelial cultures. These cells were termed "elongated". The tumour-derived cell line, Rama 25, is an essentially pure clonal line of "cuboidal" cells maintained by frequent passage, while Rama 29 is a clonal line of "elongated" cells derived from a Rama 25 culture permitted to become confluent. Dense cultures of "cuboidal" cells from Rama 25, and from the equivalent cell from normal glands, Rama 75, formed not only "elongated" cells but groups of cells with a third morphology: dark and polygonal, with many small vacuoles or "droplets" at their peripheries. These cells were termed "droplet" cells (Bennett *et al.*, 1978). Unlike the "cuboidal" cells, the patches of "droplet" cells often formed hemispherical blisters or "domes" in the cell monolayer (McGrath, 1975). The rate of "droplet" cell and "dome" formation could be rapidly accelerated with agents which induced Friend erythroleukaemic cells to differentiate (Friend *et al.*, 1974; Palfrey *et al.*, 1977), notably dimethyl sulphoxide, in the presence of insulin, hydrocortisone and prolactin (Rudland *et al.*, 1979). Clonal cell lines could be classified morphologically as one of "cuboidal", "elongated" or "fibroblastoid", as were the cells in the primary cultures. An example of the last class is Rama 27 (Bennett *et al.*, 1978).

The cultured cells were characterized by a variety of approaches. On the whole, "elongated" Rama 29 cells differed from Rama 25 cells, and more closely resembled "fibroblastoid" Rama 27 cells in many respects. Thus the ultrastructure (Bennett *et al.*, 1978); histochemical stains for the $Na^+/K^+$ ATPase (Russo *et al.*, 1977); serological stains for actin (Lazarides & Weber, 1974), myosin (Weber & Groeschel-Stewart, 1974) found in muscle and "fibroblastoid" cells; the "fibroblastoid" extra-cellular matrix glycoprotein, LETS (Graham *et al.*, 1975); the thymocyte differentiation antigen, Thy-1·1 (Letarte-Muirhead *et al.*, 1975; Dulbecco *et al.*, 1979) and the cell-surface components accessible to lactoperoxidase-catalysed iodination (Hynes & Humphryes, 1974) were similar for Rama 27 and 29, but unlike those for Rama 25 cells. However, Rama 29 cells contained a few extra features compared with normal fibroblasts, notably extracellular material resembling basal lamina, often connected to cells by hemisome-like junctions (Bennett *et al.*, 1978), and evidence consistent with the production of basement-membrane-specific (Trelstad & Slavkin, 1974) Type IV collagen (Rudland *et al.*, 1980*b*). These results suggested that the Rama 29 cells were myoepithelial-like cells (Hackett *et al.*, 1977) showing both mesenchymal and epithelial properties (Bennett *et al.*, 1978; Rudland *et al.*, 1979). Antiserum against casein (Bennett *et al.*, 1978; Warburton *et al.*, 1978) and peanut lectin (Newland *et al.*, 1979) on the other hand, reacted only with secreting epithelial cells in the mammary gland, and not with myoepithelial or mesenchymal cells; and in culture reacted weakly with "cuboidal" cells, or more strongly with cultures of "droplet" cells and "domes", but not with Rama 29 or Rama 27 cells.

We have isolated and characterized types of lining epithelial stem-cell lines from tumorous glands which can give rise to myoepithelial-like and secretory, alveolar-like cells in culture and, for Rama 25, in tumours formed in rodents (Bennett *et al.*, 1978). Morphologically similar cells are obtained from normal glands, but their precise identification awaits biochemical analysis (Rudland *et al.*, 1980*a*, *b*). Stem cells that can be converted into myoepithelial cells in the normal gland should be required during two developmental stages: firstly early in development, since myoepithelial cells can be found in the mammary rudiment before birth (Salazar & Tobon, 1974; Schlotke, 1976), and secondly when terminal end buds containing only cuboidal cells (Russo *et al.*, 1977) form alveolar buds, and then mature alveoli containing myoepithelial and secretory epithelial cells (Russo *et al.*, 1977). In support of an early role in development, Rama 25 cells, when grown on floating collagen gels (Emerman *et al.*, 1977) produce tubular structures with the epithelial cells organized round a central lumen and also 3-dimensional structures reminiscent of rudimentary mammary glands (Rudland *et al.*, 1979; Bennett & Durbin, unpublished). However, the fact that they can be induced to synthesize casein in culture supports a role for Rama 25-like cells at the later stages in development. These conclusions raise the possibility that one stem cell could perform both developmental functions.

REFERENCES

Bennett, D. C., Peachey, L. A., Durbin, H. & Rudland, P. S. (1978) *Cell*, **15**, 283.

Dulbecco, H., Bologna, M. & Unger, M. (1979) *Proc. Natl Acad. Sci. U.S.A.*, **76**, 1848.

Emerman, J. T., Enami, J., Pitelka, D. & Nandi, S. (1977) *Proc. Natl Acad. Sci. U.S.A.*, **74**, 4466.

Friend, C., Scher, W., Holland, J. G. & Sato, T. (1974) *Proc. Natl Acad. Sci. U.S.A.*, **71**, 2295.

Graham, J. M., Hynes, R. O., Davidson, E. A. & Bainton, D. F. (1975) *Cell*, **4**, 353.

Gros, R. J. (1967) *Control of Cellular Growth in Adult Organisms*. New York: Academic Press. p. 3.

Hackett, A. J., Smith, H. S., Springer, E. L. & 4 others (1977) *J. Natl Cancer Inst.*, **58**, 1795.

Hallowes, R. C., Rudland, P. S., Hawkins, R. A., Lewis, D. J., Bennett, D. C. & Durbin, H. (1977) *Cancer Res.*, **37**, 2492.

Huggins, C., Briziarelli, G. & Sutton, H. (1959) *J. Exp. Med.*, **109**, 25.

Hynes, R. O. & Humphryes, K. C. (1974) *J. Cell Biol.*, **62**, 438.

Kon, S. K. & Cowie, A. T. (1961) *Milk, The Mammary Gland and its Secretions*. New York: Academic Press. Ch. 1.

LAZARIDES, E. & WEBER, K. (1974) *Proc. Natl Acad. Sci. U.S.A.*, **71**, 2268.
LETARTE-MUIRHEAD, M., BARCLAY, A. N. & WILLIAMS, A. F. (1975) *Biochem. J.*, **151**, 685.
LYONS, R. W., LI, C. H. & JOHNSON, R. E. (1958) *Rec. Prog. Horm. Res.*, **14**, 219.
MCGRATH, C. M. (1975) *Am. Zool.*, **15**, 231.
NANDI, S. J. (1958) *J. Natl Cancer Inst.*, **21**, 1039.
NEWLAND. R. A., KLEIN, P. J. & RUDLAND, P. S. *J. Natl Cancer Inst.* (in press).
PALFREY, C., KIMHI, Y., LITTAUER, U. Z., REUBEN, R. C. & MARKS, P. A. (1977) *Biochem. Biophys. Res. Comm.*, **76**, 937.
RUDLAND, P. S., HALLOWES, R. C., DURBIN, H. & LEWIS, D. J. (1977) *J. Cell Biol.*, **73**, 561.
RUDLAND, P. S., BENNETT, D. C. & WARBURTON, M. (1979) *Cold Spr. Hb Symp. Conferences on Cell Proliferation*, Vol. 6. *Hormones and Cell Culture.* Ch. 47 (in press).
RUDLAND, P. S., BENNETT, D. C. & WARBURTON, M. (1980*a*) *1st Int. Cong. Hormones and Cancer.* New York: Raven Press (in press).
RUDLAND, P. S., BENNETT, D. C., RITTER, M. A., NEWMAN, R. A. & WARBURTON, M. J. (1980*b*) *Specific Factors in Growth and Differentiation.* New York: Raven Press (in press).
RUSSO, J., WELLS, P. A. & RUSSO, I. H. A. (1977) *Cancer Res.*, **37**, 1088.
RUSSO, J., SABY, J., ISENBURG, W. M. & RUSSO, I. H. (1977) *J. Natl Cancer Inst.*, **59**, 435.
SALAZAR, H. & TOBON, H. (1974) *Lactogenic Hormones, Fetal Nutrition, and Lactation.* London: Wiley. p. 221.
SCHLOTKE, E. (1976) *Zentralbl. Veterinaermed.* [*A*], **23**, 661.
TALWALKER, P. K. & MEITES, J. (1961) *Proc. Soc. Exp. Biol. Med.*, **107**, 880.
TRELSTAD, R. L. & SLAVKIN, H. C. (1974) *Biochem. Biophys. Res. Comm.*, **59**, 443.
WARBURTON, M., HEAD, L. & RUDLAND, P. S. (1978) *Biochem. Soc. Trans.*, **7**, 115.
WEBER, K. & GROESCHEL-STEWART, U. (1974) *Proc. Natl Acad. Sci. U.S.A.*, **71**, 4561.